# Cerebral Venous Thrombosis in a 17-Year-Old Female Patient: A Case Report

**DOI:** 10.7759/cureus.42384

**Published:** 2023-07-24

**Authors:** Maria F Casanova Rivera, Nelson B Ligua Duque, Electra A Moreno Veloz, Paullette S Casanova Rivera

**Affiliations:** 1 Department of Medicine, Universidad Católica de Santiago de Guayaquil, Guayaquil, ECU; 2 Department of Critical Care Medicine, Luis Vernaza Hospital, Guayaquil, ECU

**Keywords:** cerebral venous thrombosis, stroke, anticoagulant therapy, anticoagulation, hypercoagulable state

## Abstract

Cerebral venous thrombosis (CVT) is a rare disorder predominantly affecting young women. Clinical presentation is not specific and varies depending on the location of the thrombus. The diagnosis requires clinical suspicion with confirmation by images. Guidelines for treatment recommend heparin during the acute phase even in patients with intraparenchymal hemorrhage. It is associated with a good prognosis when diagnosed and treated promptly. We present a case of CVT and intraparenchymal hemorrhage in a 17-year-old female with severe headache, nausea, vomiting, and altered mental status. The patient was diagnosed with CVT secondary to systemic lupus erythematosus (SLE) and antiphospholipid syndrome (APS). She showed improvement after anticoagulation and corticosteroids.

## Introduction

Cerebral venous thrombosis is an uncommon disorder worldwide affecting 1/100,000 people per year representing 0.5% of all strokes [1-3]. Cerebral venous thrombosis (CVT) has a greater incidence in women of childbearing age related to hypercoagulable states [1,2,4]. The clinical manifestations of CVT depend on the location of the thrombosis and intracranial pressure development. The most common symptom is headache, reported in 90% of cases, and is reported as the only symptom in 25% of all patients [2,3].

Computed tomography and magnetic resonance images have an important role in the diagnosis, showing cerebral venous infarction in 28.6% of cases and intraparenchymal hemorrhage in 14.3% of cases. The most frequent sites involved with thrombosis are the superior sagittal sinus and the transverse sinus [5]. CVT management includes anticoagulation and treatment of the underlying cause when established [6]. The majority of patients present favorable outcomes with 80% of them recovering completely [3].

Antiphospholipid syndrome (APS) is an autoimmune disease defined by laboratory and clinical findings associated with arterial or venous thrombosis or obstetrical events [7-9]. The patients commonly present venous thromboembolism usually affecting the legs, with a prevalence of 31.7%-38.9%, but cerebral venous thrombosis is rare with a prevalence of 0.7% [5,10]. APS is usually associated with lupus erythematosus systemic [7,10]. CVT occurs in 0.37% of systemic lupus erythematosus (SLE) cases [11].

## Case presentation

A 17-year-old female presented to the emergency room with chief complaints of severe left-sided headache accompanied by nausea and vomiting developed over the preceding 48 hours and confusion and somnolence developed 24 hours before admission. The history was obtained from her mother due to patient status who referred that the patient was diagnosed with white coat hypertension five years ago and history of polyarticular joint pain and malar rash 10 months before admission, therefore started taking prednisone occasionally without being prescribed. The patient had no history of oral contraceptive use, pregnancy, and thrombophilia. A family history of thrombophilia was denied. On examination, the patient was afebrile, with blood pressure of 168/122 mmHg, 89 beats per minute, and oxygen saturation (SpO_2_) of 96% on room air. She was somnolent with a Glasgow Coma Scale (GCS) of 13 (eye-opening 3, verbal 4, motor 6), without motor or sensory impairment. Laboratory findings revealed a hemoglobin of 8.6 g/dL (normal range: 12.1-15.1), hematocrit 26 (normal range: 36-48), platelets 213,000 (normal range: 150,000-450,000), white blood cells 14,810 (normal range: 4,500-11,000), neutrophils 76% (normal range: 40%-60%), lymphocytes 13% (normal range: 20%-40%), partial thromboplastin time (PTT) 25.6 (normal range: 25 to 35 seconds), prothrombin time (PT) 11.6 (normal range: 11 to 13.5 seconds), international normalized ratio (INR) 0.96 (normal range: 0.8 to 1.1), blood urea nitrogen 86 mg/dl (normal range: 5-20), creatinine 1.53 mg/dL (normal range: 0.7-1.3), electrolytes within normal limits, positive lupus anticoagulant, anti-Ro, anti-Sm, and anti-double-stranded DNA (dsDNA), antinuclear antibody (ANA) 953.5 (positive ANA titer >55 U/ml), anti-beta-2 glycoprotein 1 (B2GP1) IgG antibodies 62.01 (>10.0 U/mL positive), anti-beta-2 glycoprotein 1 (B2GP1) IgM antibodies 14.95 (>18.0 U/mL positive), cardiolipin antibody IgM 16.9 (>45.0 U/mL positive), serum complement C3 of 67 (normal range: 80-178), and C4 of 6.8 (normal range: 15-52). Immune serology findings were consistent with systemic lupus erythematosus and antiphospholipid syndrome.

**Figure 1:**
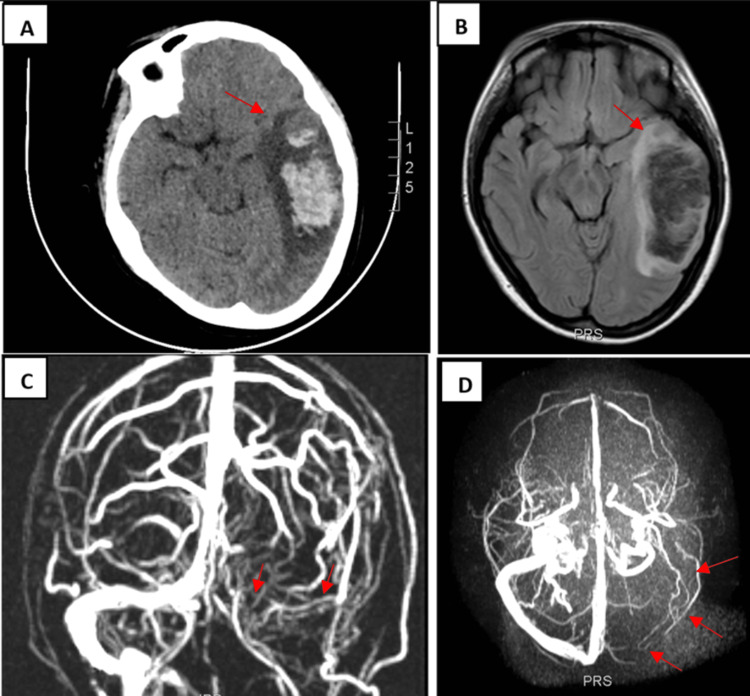
Non-contrast CT scan, FLAIR, and MRV images. Non-contrast CT scan of the head showed a left parietotemporal intraparenchymal hemorrhage with surrounding edema (red arrow) (A). FLAIR showed intraparenchymal hemorrhage with edematous changes (red arrow) (B). MRV images showed an absence of filling of the left transverse sinus (red arrows) (C, D). CT: computed tomography, FLAIR: fluid-attenuated inversion recovery, MRV: magnetic resonance venography.

Computed tomography (CT) scan of the head was obtained and showed a hyperdense lesion suggesting left parietotemporal intraparenchymal hemorrhage with surrounding vasogenic edema and partial collapse of the left side ventricular system, without midline displacement. Fluid-attenuated inversion recovery (FLAIR) showed broad high-intensity lesions with edematous changes consistent with left parietotemporal intraparenchymal hemorrhage. Magnetic resonance venography (MRV) was obtained for confirming the diagnosis of CVT; it showed an absence of filling of the left transverse sinus. Based on image findings, the diagnosis of left transverse venous sinus thrombosis was made (Figure 1).

**Figure 2:**
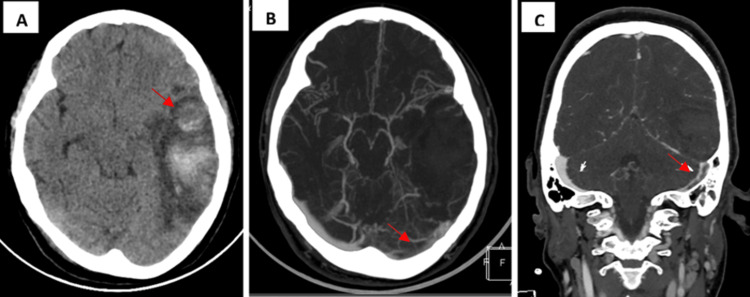
Follow-up CT scans eight days after admission and CT angiography. Follow-up CT scans eight days after admission showed a reduction of the dimensions of the left parietotemporal intraparenchymal hematoma (red arrow) (A). CT angiography showed a partially recanalized left transverse sinus (red arrow) (B, C). CT: computed tomography.

Given progressive mental status deterioration, she was intubated and ventilated and presented with a hypertensive crisis. She was initiated on intravenous antihypertensive medication, methylprednisolone, hydroxychloroquine, calcitriol, erythropoietin, anticoagulation with heparin, and received cyclophosphamide. Funduscopic examination was normal. Electroencephalogram showed no abnormalities. Two days after admission, the patient was anuric and needed renal replacement therapy due to acute kidney failure due to systemic lupus erythematosus; laboratory exam revealed blood urea nitrogen 147 mg/dl (normal range: 5-20) and creatinine 2.91 (normal range: 0.7-1.3). Follow-up CT scans were performed eight days after admission and showed a reduction of the dimensions of the left parietotemporal intraparenchymal hematoma and perilesional edema with a partially recanalized left transverse sinus in CT angiography (Figure 2).

Ten days after the patient was extubated without complications, the intravenous antihypertensive medication was changed to oral antihypertensive drugs. Her symptoms completely improved and was discharged on day 20. The cause of venous sinus thrombosis was established to be secondary to systemic lupus erythematosus and antiphospholipid syndrome.

## Discussion

CVTs are rare; 80% of CVT cases are presented in young women with hypercoagulable states. Multiple risk factors are strongly associated with CVT presentation such as pregnancy, puerperium, hormone replacement therapy, oral contraceptive use, and thrombophilia. Thrombophilia has been established as the most common cause [1,2,4,12]. However, initial thrombophilia screening is not recommended in patients with a low probability of this pathology [12]. In 12.5% of cases, no cause can be established [2].

Patients with SLE and APS may present CVT; however, it is an unusual clinical manifestation [5,11-13]. Signs and symptoms rely upon the location of the thrombus; clinical manifestations include headaches reported in 90% of patients, focal deficit in 40%, altered mental status in 20%, nausea and vomiting in 38.1%, and seizures in 40% [6,14]. The diagnosis could be challenging and requires clinical suspicion confirmed by images showing complete or partially obstructed cerebral veins [4,12,14,15]. The most commonly affected sites are the superior sagittal sinus in 65% of patients and the transverse sinus in 60.5% [15]. Computed tomography and magnetic resonance images show a greater prevalence of cerebral venous infarction followed by intracerebral hemorrhage [4,12,14,15].

Our patient presented with headache, nausea, vomiting, and altered mental status which increased concern for intracranial hypertension; therefore, computed tomography scan was performed with posterior MRV for confirming the diagnosis of CVT. Previous history of polyarticular joint pain and malar rash guided the diagnosis of autoimmune disease, and it was confirmed by positive lupus anticoagulant, anti-beta-2 glycoprotein 1 (B2GP1) IgG antibodies, anti-Ro, anti-Sm, and anti-DNA. Due to the laboratory findings and clinical manifestations, we were able to establish that the CVT was developed secondary to systemic lupus erythematosus and antiphospholipid syndrome.

European and American guidelines indicate that anticoagulation with heparin is the recommended treatment of choice even in the presence of intraparenchymal hemorrhage. Lower molecular weight heparin (LMWH) has shown a lower risk of severe hemorrhagic complications and mortality versus unfractionated heparin (UFH). Vitamin K antagonists are recommended for three to 12 months to prevent CVT recurrence and other thrombotic events. Steroids are not associated with CVT improvement. Antiepileptic drugs are suggested in patients with acute CVT with supratentorial lesions and seizures to prevent early recurrent seizures. Proper treatment for autoimmune disease is necessary to avoid recurrence and future complications [1,3,12].

Approximately 80% of patients present complete recovery. Higher mortality and risk of recurrence are associated with cases without the risk factors described above [1]. Our patient received intravenous methylprednisolone 500 mg for three days, prednisone 60 mg daily, hydroxychloroquine 200 mg daily, calcitriol daily, and cyclophosphamide 500 mg every 15 days for two occasions for her autoimmune disease, and heparin for CVT. After 20 days of hospitalization, she had her symptoms improved with hematoma reabsorption.

## Conclusions

Cerebral venous thrombosis is a rare condition mostly present in young women associated with hypercoagulable states. In a young patient with no history of hormone replacement therapy, oral contraceptive use, pregnancy, or puerperium, thrombophilia should be considered and screening must be performed when a high probability of thrombophilia is present based on associated family history or clinical manifestations. Clinical manifestations are variable, and clinical suspicion must be confirmed with images. Anticoagulation with heparin is the treatment of choice even in patients with intraparenchymal hemorrhage. CVT has a good prognosis when diagnosed and treated early.
